# Natural radioactivity and element characterization in pit lakes in Northern Sweden

**DOI:** 10.1371/journal.pone.0266002

**Published:** 2022-03-31

**Authors:** Rimon Thomas, Juan Mantero, Carlos Ruiz Cánovas, Elis Holm, Rafael García-Tenorio, Eva Forssell-Aronsson, Mats Isaksson

**Affiliations:** 1 Department of Medical Radiation Sciences, Institute of Clinical Sciences, Sahlgrenska Academy at University of Gothenburg, Gothenburg, Sweden; 2 Department of Applied Physics II, ETSA, University of Seville, Seville, Spain; 3 Department of Earth Sciences & Research Center on Natural Resources, Health and the Environment, University of Huelva, Huelva, Spain; 4 Spanish National Accelerator Centre (CNA), University of Seville, Seville, Spain; 5 Department of Medical Physics and Biomedical Engineering, Sahlgrenska University Hospital, Region Västra Götaland, Gothenburg, Sweden; University of Western Australia, AUSTRALIA

## Abstract

Northern Sweden has been the object of intense metal mining in the last decades producing several water-filled open-pits, or pit lakes. Most of these pit lakes have been limed to maintain a good water quality and to prevent generation of acidic water that could leach the exposed rocks and release metals into water. The aim of this work was to examine the concentration of stable elements and naturally occurring radionuclides in water and sediment samples from pit lakes originating from non-uranium mining activities in Northern Sweden. Surface water and surface sediments were collected from 27 pit lakes in Northern Sweden. Water quality parameters, concentration of stable elements and radionuclides were measured by a water probe, ICP-MS and XRF, and alpha and gamma spectrometry, respectively. Furthermore, a multivariate statistical analysis (PCA) was performed on the water samples and sediments. In general, the quality of the surface water was good, but some lakes had low pH values (2.5–5.7), and high concentrations of Fe (up to 200 mg/L) and other metals (e.g. Zn, Cu). When relating the metal concentrations in sediments in pit lakes with the concentration found in natural lakes, some sites had relatively high levels of Cu, As, Cr and Pb. The activity concentration of ^210^Po, and U and Th isotopes in water and sediment samples were at environmental levels, as was the ambient dose equivalent rate at these sites (range 0.08–0.14 μSv/h).

## Introduction

Sweden is one of the most important producers of ores and metals of the European Union; it is by far the biggest producer of iron ore and is also among the leading producers of copper, zinc, lead, gold and silver. Mining activities, mainly open-pit mining, implies large quantities of generated mining wastes. Open-pit mining is mainly used when ore deposits are found near the surface and lead to the formation of large voids. Among the active mines in Sweden the annual production of mining waste, mainly waste rock and tailings, is more than 100 million tons (as of 2017) [1]. Waste rock backfilling is one of the most commonly employed methods for land rehabilitation of open-pit mines, however, the amount of waste rock produced is commonly too small to allow effective reclamation by backfilling. Thus, the open-pit remains void, developing into a pit lake [2]. The mining activities in Sweden have generated more than 2700 mining sites, of which only 15 remain active today, most of them in the Northern part of Sweden [3]. In contrast to pit lakes in Southern Sweden, which are mainly former quarries and located in densely populated areas with a high recreational value, pit lakes in Northern Sweden are mostly former metal mines and found in sparsely populated areas with high ecological value [4].

The geology in Northern Sweden is the result of a complex geodynamic evolution including repeated extensional and compressional tectonic regimes and associated magmatic and metamorphic events [5]. The main crustal growth of the Baltic Shield occurred during the Svecofenian orogeny, simultaneously to the accretion of the Karelian Craton. The Baltic Shield is divided in three Provinces. Volcanosedimentary active continental-arc and volcanic-arc rocks mainly constitute the North-Svecofenian province while the Central-Svecofenian province is a deep marine sedimentary area of the Bothnia Basin. The South-Svecofenian province is composed of island-arc volcanics [6].

The presence of sulfides in these deposits may lead to acid mine drainage (AMD) processes, one of the main causes of water deterioration worldwide [7]. The assessment of the water quality at reclaimed lakes affected by AMD is a critical step in evaluating the performance of remediation measures and the implementation of improvements. In addition to mining, other anthropogenic activities may also have a significant impact on lake waters. This is the case of atmospheric deposition of acidifying compounds of industrial origin, which commonly leads to severe acidification of lakes and streams [8]. Acidification of surface waters, caused by both AMD and atmospheric deposition of acidifying compounds, has been of great concern in the past. For this reason, liming has, for example, been used in Sweden since the 1970’s to limit acidification of surface waters [9]. It is estimated that around 8000 lakes have been limed at least once and about 20 million €/y has been devoted to liming of surface waters [10]. The main aim for liming was to keep alkalinity above 0.05 meq/L and pH above 6.0. Liming causes the precipitation of metals from the water column to the lake sediments by increasing the pH. However, if re-acidification prevails, re-solubilization of metals can occur, leading to increased metal concentrations in the water column. Therefore, the metal content in environmental reservoirs of such lake waters should be monitored in order to ascertain the effectiveness of remediation measures.

The radiometric characterization of pit lakes has historically received less attention than investigations of metals and organic compounds. Previous studies on pit lakes affected by AMD processes with low pH (2,2–2,7) in the Iberian Pyrite Belt have shown enhanced levels of naturally occurring radionuclides [11]. Conversely, in alkaline pit lakes (pH 9.2–9.6), enhanced levels of ^238^U and ^234^U (up to 2000 mBq/L and 8600 mBq/L, respectively) have been reported [12]. As a reference, typical values in superficial water are 11–32 mBq/L and 13–40 mBq/L for ^238^U and ^234^U, respectively [13]. In case of alkaline pH values and elevated bicarbonate concentrations, oxidized surface water favors the complexation and mobilization of uranium as uranyl-carbonate complex [14], while the predominant species in acidic, oxygenated waters are the uranyl ion and uranyl-sulfate complex [15]. Thus, the potential increase of concentration of radionuclides in pit lakes caused by both pyrite oxidation and liming activities should be further studied.

Mantero et al. [4] recently performed a study of radionuclide and elemental concentrations in pit lakes in Southern Sweden, where increased concentrations of U isotopes were found. Previous studies of various lakes in Northern Sweden have dealt with concentration of stable elements and the impact of liming [16–18]. However, radionuclide concentrations have received less attention. Therefore, the main aim of this work was to perform a screening study of the many pit lakes that can be found in northern Sweden by measuring radionuclides and metals in surface water and surface sediment. A comparison between limed and non-limed pit lakes on the metal and radionuclide concentration has also been made, however, the extent of liming (amount and how often) are not known.

## Material and methods

### Pit lake locations

The sampling was carried out in July 2015 and April 2016, and included 17 different mining sites in Northern Sweden, denoted N1 to N17, with the additional letters A, B and C denoting each pit lake if several occurred at the same site. The sampled sites are located in areas with low ^238^U and ^232^Th concentration in bedrock, around 25 Bq/kg [19]. A brief description of the sampled sites and coordinates of the pit lakes can be found in S1 Table in S1 File (along with pictures of the lakes) in the supplementary data file. The main metals mined at these sites were Cu, Zn, Pb and Au. Most of the mines closed during the 80 and 90’s; the oldest dates back to 1947 (Site N7) and the youngest is from 2001 (Site N8). The ambient dose equivalent rate was measured with a dose rate meter (SRV2000, Rados), at 1 m height at several points around each pit lake.

### Sample collection

Surface water (top 0.5 m) was collected once from each pit lake (n = 27) in 5L polyethylene containers (Fisher brand™, Fisher Scientific). The containers were first rinsed with distilled water and then with water from the pit lake before sample collection. The samples were collected from one point of the lake, usually near the shoreline (where the lake was accessible) and thus the collected water might not represent the whole lake. Since all pit lakes included in this study had unknown activity concentrations of naturally occurring radionuclides and most had unknown data on metal concentrations, raw water was the focus in this study. This is to avoid overlooking pit lakes with relatively high concentration of radionuclides and metals e.g. bound to humic acids and colloids, and to increase the knowledge in what can be considered as raw water (total concentration). Furthermore, the total concentration is relevant to the exposure of living organisms in the water. Therefore, the acidification was done in connection with the sampling, prior to any filtration since total concentration was of interest. The acidification was achieved with a few drops of concentrated (65%) HNO_3_ acid to reach a pH value < 2.

Water samples analyzed by alpha spectrometry were in most cases not filtered since the collected water was mostly free from visible debris such as leaves and broken-down plants. If such debris was observed, the water was filtered with a coarse filter of pore size 35–40 μm. Water samples analyzed by an inductively coupled plasma mass spectrometer (ICP-MS) were always filtered with a filter paper of 35–40 μm pore size (filtration was mandatory as part of the methodology at the facility).

Collection of sediment from accumulation sites, such as bathymetric low points, was in general not possible for these pit lakes. Most pit lakes are remotely located or have too steep walls, which made it impossible to use a boat to reach the deepest location. Therefore, surface sediments, top 1 cm, were collected from the shoreline at water depths of about 1 m (n = 19). When possible, sediments were collected from different areas along the shoreline and combined to one composite sample. The sediments were homogenized, dried at 80°C, milled, and finally sieved to 1 mm size for the determination of elemental composition by X-ray fluorescence and radionuclide concentration by alpha and gamma spectrometry.

### Analytical methods

Water quality parameters, such as temperature (T) and specific conductivity (SC) (corrected to 25°C) were measured with a water probe (Professional Plus, YSI) submerged in the lake at the same site and depth where water was collected. Elemental composition (major and trace elements) was determined in water samples by ICP-MS, and in sediment by X-ray fluorescence (XRF). Activity concentration of ^238^U, ^234^U, ^232^Th, ^230^Th and ^210^Po in water were measured by alpha spectrometry, and gamma emitting radionuclides in the ^238^U and ^232^Th series in sediments were measured by gamma spectrometry. For the latter, the radionuclides included were ^210^Pb, ^234^Th and ^226^Ra (determined by secular equilibrium using ^214^Pb and ^214^Bi), ^228^Ra (through ^228^Ac) and ^228^Th (through ^212^Pb, ^212^Bi and ^208^Tl).

The ICP-MS system (Agilent 7500c, Agilent Technologies) was calibrated with a standard solution containing 18 elements from Be to U and provided a detection limit around 30 ng/L for most elements. Using 0.1 g of sample the WD-XRF detector system (Axios, Malvern Panalytical) gave uncertainties of around 10% and detection limits between 1 and 100 ppm for most elements. For alpha spectrometry and for solids, about 1 g of sample was digested with HF acid and aqua regia through microwave assisted digestion, for water samples about 500 mL were used. The tracers used to calculate the activity concentration were ^232^U, ^229^Th and ^209^Po. Separation between the radionuclides was performed through liquid-liquid-extraction with tri-n-butyl phosphate and a chromatography column (UTEVA, TrisKem). The fractions were then plated on metal discs and measured with ion-implanted silicon charged particle detectors (Alpha Ensemble, ORTEC). The detection limits were about 0.5 Bq/kg for solids and 0.5 mBq/kg for water samples. Samples analyzed by gamma spectrometry were packed in 35 mL cylindrical containers. The high purity germanium detector (XtRa, CANBERRA) provided an energy resolution of 1.76 keV at 1332 keV and a detection limit ranging from 2 to 10 Bq/kg for most radionuclides. The detector was calibrated using the reference materials IAEA-RGU and IAEA-RGTh, which contain the same radionuclides as those included in this study. A more detailed description of the analytical techniques and radiochemical procedure have been published along with quality control (QC/QA) tests applied for all these techniques [20, 21].

### Calculated quantities

The concentration of metals in sediments can be used in the diagnostic tool proposed by Håkanson (1980) [22] in order to estimate the contamination in pit lakes. With this tool a parameter defined as the degree of contamination (C_d_) can be calculated, which is based on eight preindustrial reference values of polychlorinated biphenyl (PCB) (0.01), Hg (0.25), Cd (1.0), As (15), Cu (50), Pb (70), Cr (90) and Zn (175) with concentrations in parenthesis given in ppm, see Eqs 1 and 2. These reference values are average concentrations for the substances in the 0–1 cm sediment layer from 50 lakes in Europe and America. Only the metals detected by XRF were included in this calculation, not PCB, and thus a modified version of this diagnostic tool was used (*i* < 8). Although this tool was created for natural lakes which exhibits some differences in size and limnology to the pit lakes included in this study, it could be considered a good approach to examine their metal concentrations.

$$C_{f}=\sum_{i=1}^{n}\frac{C_{0-1}^{i}}{C_{ref}^{i}},$$ (1)

$$C_{d}=\sum_{i=1}^{n}C_{f}^{i},$$ (2)

where $C_{0-1}^{i}$ is the concentration of the i-th substance, $C_{ref}^{i}$ is the preindustrial reference value, and $C_{f}^{i}$ is the contamination factor (C_f_) of the i-th element. Håkanson assigns four risk categories for $C_{f}^{i}$, low (< 1), moderate (1–3), considerable (3–6), and very high (> 6). Similarly, for C_d_: low (< 8), moderate (8–16), considerable (16–32) and very high (> 32) degree of contamination. However, threshold values for C_d_ were adjusted to the number of elements included in the calculation. Elemental composition of 19 sediment samples were included in Eqs 1 and 2. to assess both degree of contamination (C_d_) and contamination factors (C_f_) in each site.

The R_S,W_ ratio was calculated for each element through its concentration in surface sediment to its concentration in (raw) surface water, which is comparable to the distribution coefficient (K_d_) with two main differences. First, the concentration in surface water reflects raw water concentration (no filtration) for alpha spectrometry, and coarse filtration (35–40 μm) for ICP-MS analysis, as opposed to 0.45 μm filtration (soluble fraction) used in determination of K_d_. Secondly, elements with high concentration in surface water were included. R_S,W_ thus reflects the total effect of physical and chemical processes.

### Data analysis

Statistical treatment of data was performed by the software IBM® SPSS Statistics (version 28), where linear regression (stepwise method), Spearman’s correlation (two-tailed test) and Wilcoxon signed-rank test (α = 0.05) were used.

## Results and discussion

### Water quality and elements in surface water

**Fig 1:**
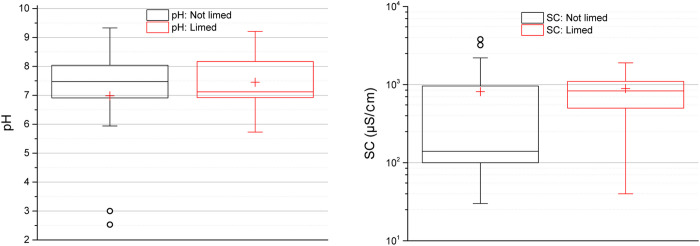
Water quality parameters pH and specific conductivity (SC) in surface water. Limed pit lakes (n = 13) in red, and non-limed pit lakes (n = 14) in black. The height of the box shows the interquartile range, which contains 50% of the values, the horizontal line inside the box shows the median value and the red cross denotes the mean value. The whiskers are lines that extend from the box to the highest and lowest values excluding outliers (o). Outliers represent those data with values being 1.5 higher or lower than the height of the box. Detailed data are given in supplementary data file.

The median pH value in surface water from 27 studied lakes was 7.3 (interquartile range of 6.9–8.1; range 2.5–9.3) and median specific conductivity (SC) was 500 μS/cm (interquartile range of 110–1100 μS/cm; range 34–3800 μS/cm), Fig 1. The values for SC are typical for groundwater in Sweden [23]. However, outlier values were also observed, defined as 1.5 higher or lower than the interquartile range which contains 50% of the values. The lowest pH value of 2.5 was found in one lake (site 9), which also had the second highest SC value of 3200 μS/cm. The acidic water is likely causing an increase in leaching of metals from lake walls and sediment and promoting their solubility, thus increasing the conductivity. The highest SC of 3800 μS/cm and the second lowest pH value of 5.9 were found in a shallow lake (site 11B) near a former Zn and Cu mine. Lakes at both sites 9 and 11B had high concentration of metals in surface water. In contrast, another former Cu and Au mine (site 12), which had been limed had a pH of 9.2 and SC of 700 μS/cm, showing an improved water quality with much lower concentration of metals in surface water.

**Fig 2:**
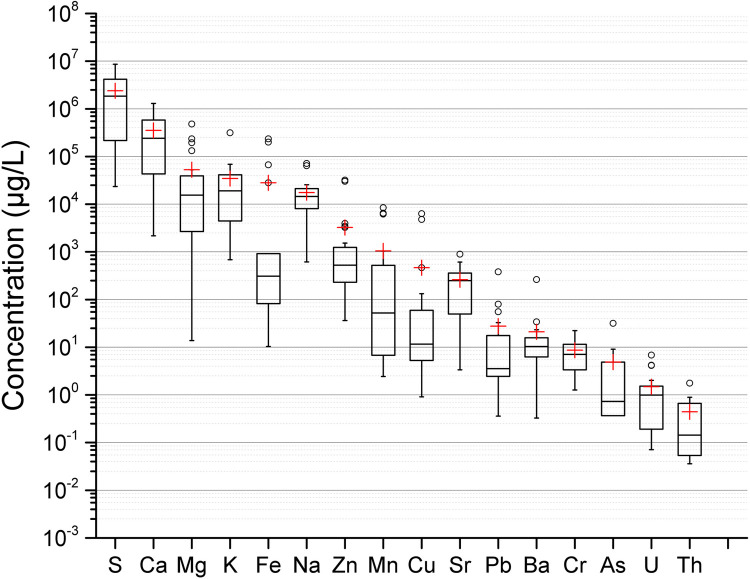
Major and trace metals in μg/L in surface water from 27 pit lakes. The height of the box shows the interquartile range, which contains 50% of the values, the horizontal line inside the box shows the median value and the red cross denotes the mean value. The whiskers are lines that extend from the box to the highest and lowest values excluding outliers (o). Outliers represent those data with values being 1.5 higher or lower than the height of the box. Detailed data are given in supplementary data file.

Fig 2 shows the elemental concentrations of lake surface water for 27 pit lakes. The concentration of metals in water depends on natural factors such as air-water-rock interactions and anthropogenic activities (e.g. industrial and liming). The study area has been intensively affected by mining due to its mineral richness. Sulfur and Ca were the most abundant elements, with interquartile ranges of 220–4200 mg/L and 40–580 mg/L, respectively. Such high values of S may be caused by the occurrence of sulfide minerals associated with local geology and to a lesser extent with air emissions.

The median concentrations of Mg, K and Na were 14 mg/L, 17 mg/L and 14 mg/L, respectively. In some lakes, Mg concentration was very high, and as in the case of Ca, may be mainly related to liming, while high Na and K values would result from the weathering of aluminosilicates of host rocks. These values are noticeably higher than those reported by Borg [16] in forest lakes of Northern Sweden with average concentration values ranging from 0.41 mg/L of K to 2.9 mg/L of Ca. Apart from an increase in Ca concentration due to liming, cation exchange can also take place, where Ca can displace adsorbed cations from the sediment to the water, further enhancing the concentration of various metals and radionuclides [24, 25]. In addition, weathering of host rock containing aluminum silicates will also release metals such as Na and K into the water.

The highest concentrations were found for Fe (240 mg/L) at Site 9 (pH 2.5), which is a lake currently used for mineral reprocessing. This site also had the highest concentrations of Zn (31 mg/L), Cu (6.4 mg/L) and Pb (0.38 mg/L). Site 11B (a shallow lake) was likely created due to changes in water table level caused by the nearby pit lake (site 11A, 50 m away). This shallow lake had the highest concentrations of Mn (8.4 mg/L) and S (8600 mg/L), and also high Fe concentration (200 mg/L).

It should be pointed out that since the sampling was partly carried out in June (i.e. summer), the surface water collected from pit lakes with sufficient depth to allow the stratification of the water, might not represent the whole lake.

**Fig 3:**
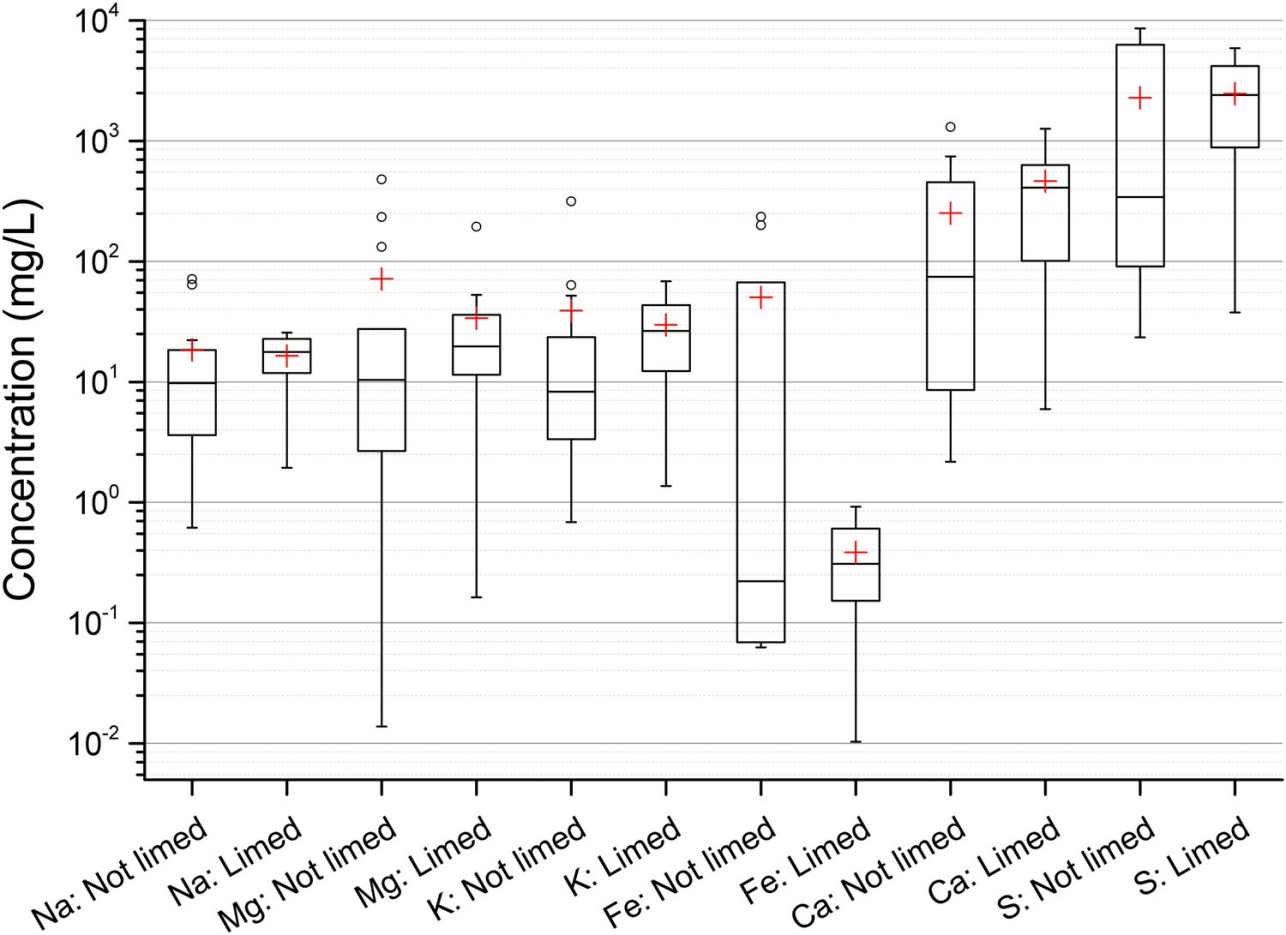
Comparison of the concentration of Na, Mg, K, Fe, Ca and S in surface water between limed and non-limed pit lakes. The height of the box shows the interquartile range, which contains 50% of the values, the horizontal line inside the box shows the median value and the red cross denotes the mean value. The whiskers are lines that extend from the box to the highest and lowest values excluding outliers (o). Outliers represent those data with values being 1.5 higher or lower than the height of the box. Detailed data are given in supplementary data file.

Among the 27 pit lakes, 13 have been limed while the remaining 14 have not been limed. Since both groups (limed and not limed) included similar types of mines (sulfide ore with Cu and Zn mineral deposits) a comparison between both groups for the concentration of Na, Mg, K, Fe, Ca and S is shown in Fig 3. The differences in concentration of Ca and Fe between limed and non-limed lakes where not significant with a Wilcoxon signed-rank test with α = 0.05. However, it is known that liming can increase the concentration of Ca in the surface water and precipitate Fe-hydroxides (reducing the Fe concentration in the water) [9, 18].

### Radionuclides in surface water

**Fig 4:**
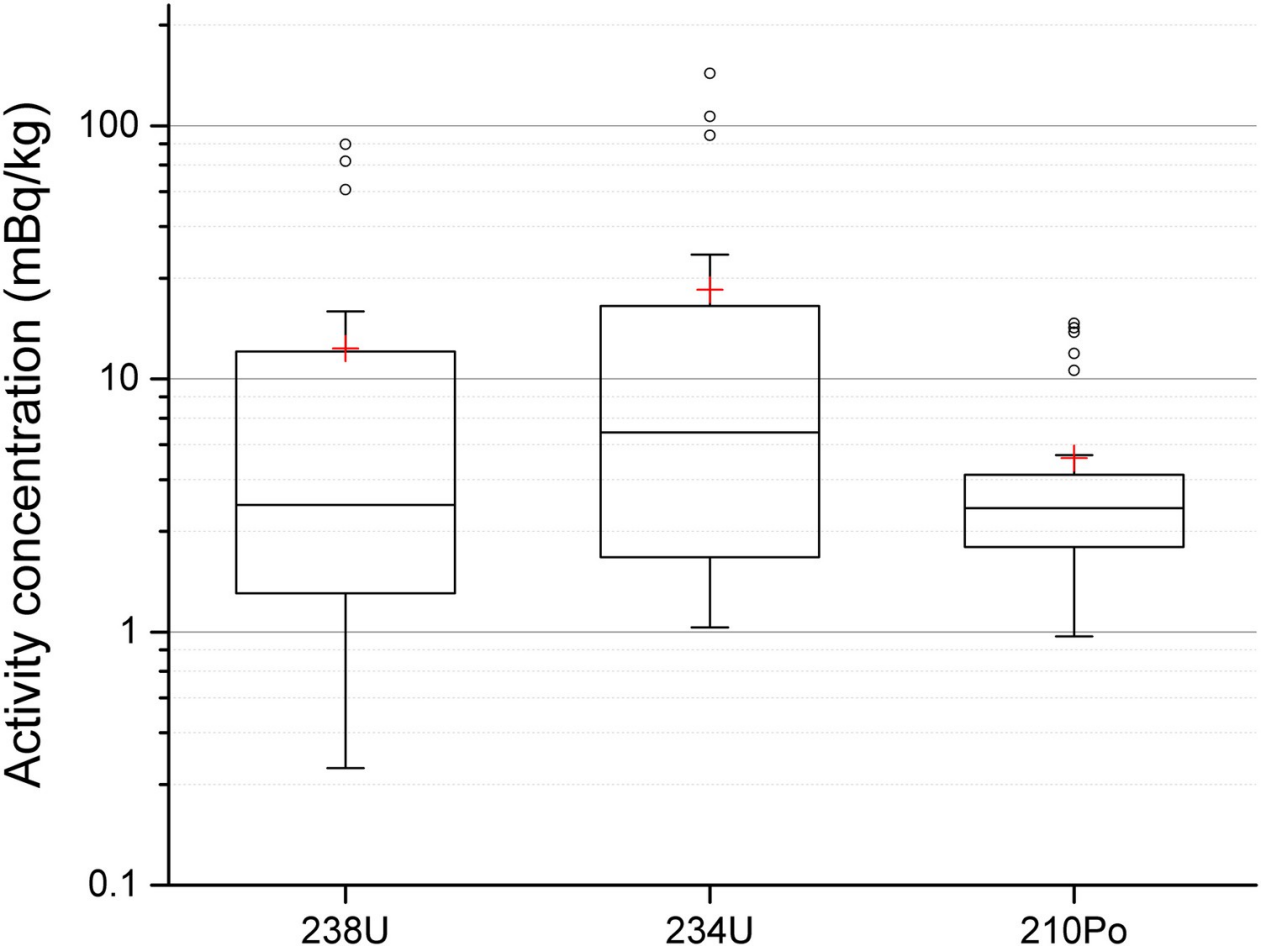
^238^U, ^234^U and ^210^Po activity concentration in surface water from 27 pit lakes. Results were obtained by alpha spectrometry. The height of the box shows the interquartile range, which contains 50% of the values, the horizontal line inside the box shows the median value and the red cross denotes the mean value. The whiskers are lines that extend from the box to the highest and lowest values excluding outliers (o). Outliers represent those data with values being 1.5 higher or lower than the height of the box. Detailed data are given in supplementary data file.

In surface water, the activity concentration of ^238^U was 0.3–85 mBq/kg (14 ± 24, mean± SD), 0.3–160 (16 ± 36) for ^234^U, 1–16 (5 ± 5) for ^210^Po, Fig 4. The concentration of ^238^U in groundwater in Sweden was mostly below 5 μg/L (49% of totally 2875 sampled sites) [23], and the average concentration in water was about 0.9 μg/L from a survey of 26 European countries [26]. These values correspond to about 62 mBq/L and 11 mBq/L, respectively. In comparison, similar concentration of U (^238^U) in surface water was found in the pit lakes in Northern Sweden. However, three lakes showed slightly higher activity concentration of ^234^U and ^238^U (site 8, 9 and 17) than the other pit lakes.

The ambient dose equivalent rate measured at all sites varied from 0.08 to 0.14 μSv/h, which can be considered to be background levels in Sweden. These rates are slightly lower than those observed in Southern Sweden, ranging from 0.06 to 0.37 μSv/h [4]. The three lakes with the highest concentration of U in surface water did not show a high(er) ambient dose equivalent rate at the sites. For example, site 8, which is a former Au mine had an activity concentration of ^238^U in surface water of 85 mBq/kg (the highest measured concentration) and a dose rate of 0.08 μSv/h (lowest measured value), while site 2, also a former Au mine, the activity concentration of ^238^U in surface water was 6.6 mBq/kg and dose rate was 0.13 μSv/h. Thus, the measurement of dose rate cannot be used as a guidance to locate lakes with higher activity concentrations if the concentration of U in the bedrock is relatively low, which was the case for the sampled sites.

^234,238^U and ^210^Po were detected in all pit lakes when sample mass of 500 g was used, however, Th isotopes were more difficult to detect due to the low activity concentration in surface water. Only one sample (site 9) had activity concentration of both ^232^Th and ^230^Th above the minimum detectable activity (MDA) which ranged from 0.1 to 1.2 mBq/kg. A larger sample (1 kg) would have been more preferable for detection of Th isotopes. Compared with pit lakes from Southern Sweden [4], similar activity concentrations were found for Th isotopes in surface water. The two pit lakes in Southern and Northern Sweden which had the lowest measured pH value also had the highest activity concentration of Th isotopes in surface water. This was also observed in a previous study of acidic pit lakes from the Iberian Pyrite Belt (geographical area with high sulfide deposits), where ^232^Th and ^230^Th activity concentrations ranged between 18–120 mBq/kg and 14–390 mBq/kg, respectively [11].

It is known that organic and inorganic ligands are responsible for increasing the solubility of Th in water, where one of the dominating species at low pH are the Th(SO_4_)_2_ complex [27]. In the two pit lakes mentioned above (in Southern and Northern Sweden), the former had a pH of 5.9 and the latter 3.0. The sulfur concentration (assumed to be present as sulfate) in surface water in the northern pit lake was 10 times higher, which together with a lower pH should facilitate a higher solubility of Th. However, both had a ^232^Th concentration of about 8.8 mBq/kg, while ^230^Th was twice as high in the southern pit lake, a consequence of the high ^238^U concentration (~ 1 Bq/kg). Thus, when comparing the concentration of Th isotopes in surface water between different sites one needs to consider many factors, apart from pH and possible ligands available for complexation, also the inherent concentrations of radionuclides at the site (bedrock). One suggestion would then be to take the ratio of ^232^Th to ^238^U to limit this confounding effect caused by naturally occurring radioactivity.

Furthermore, there was a linearity between the concentration of the isotopes of the same radionuclide in surface water, with R^2^ > 0.96 for ^238^U-^234^U and for ^232^Th-^230^Th in the pit lakes in Northern Sweden. This observation for the Th isotopes, which originate from two different decay series, supports the fact that both decay chains need to be studied for the interpretation of activity concentration of Th isotopes in water.

Interestingly, the pit lakes with the highest concentration of ^238^U did not demonstrate the highest concentration of ^210^Po. The ^210^Po-to-^238^U concentration ratio ranged from 0.03 to 44 (3.5 ± 9.3, mean ± SD), and the ^234^U-to-^238^U concentration ratio ranged from 0.74 to 2.3 (1.5 ± 0.4). These disequilibria are similar to those found in pit lakes in Southern Sweden [4], with U ratios ranging from 0.77 to 2.0 (1.3± 0.2). For pit lakes in Morocco with alkaline water, the ^234^U-to-^238^U concentration ratio was 4.3–5.4 [12], and in acidic pit lakes in Spain the ratio was 1.6–4.5 [11]. Ratios higher than unity is commonly found in groundwater [28] and has been reported to be as high as ten [29].

**Fig 5:**
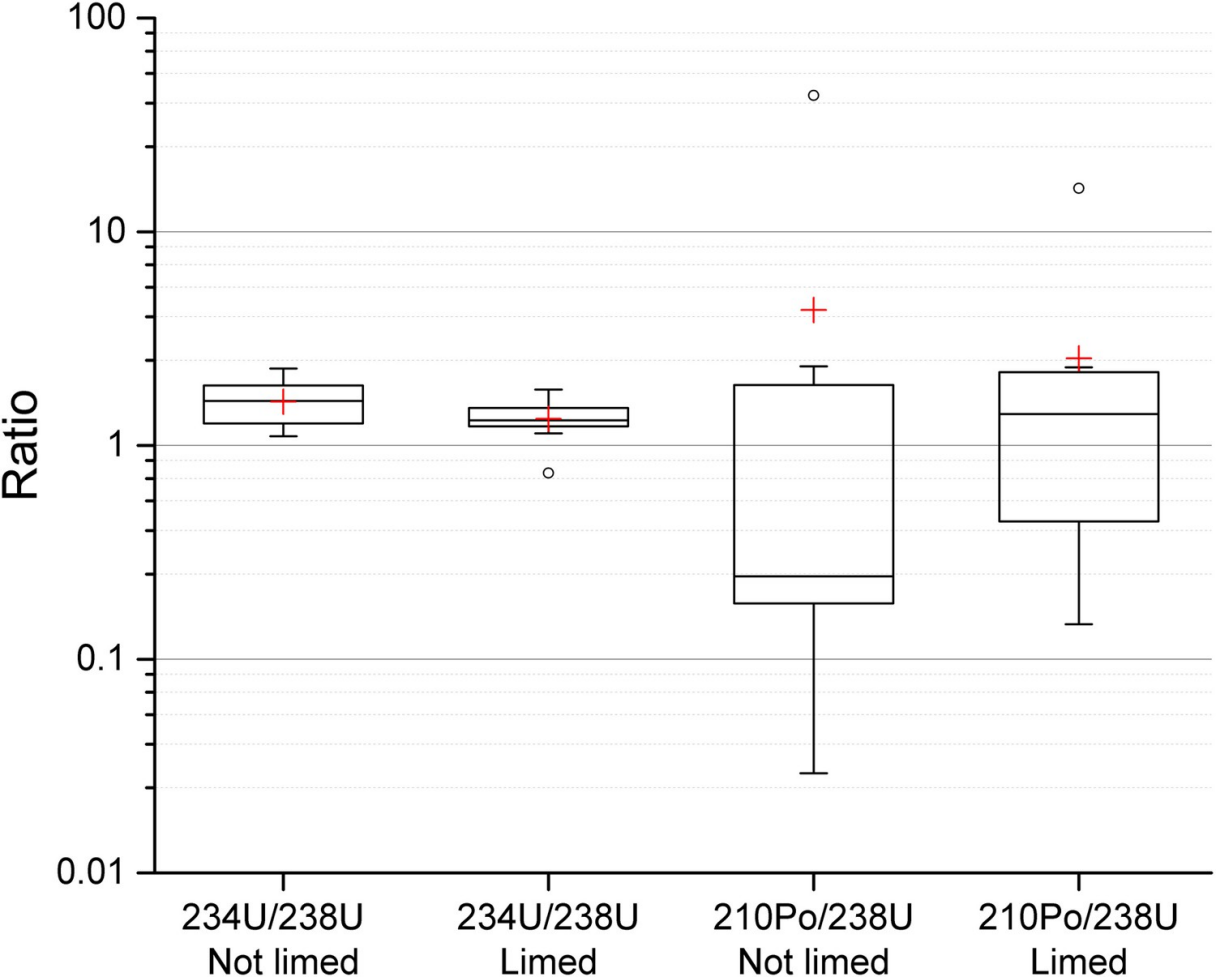
^234^U-to-^238^U and ^210^Po-to- ^238^U activity concentration ratios in surface water from limed and not limed pit lakes. The height of the box shows the interquartile range, which contains 50% of the values, the horizontal line inside the box shows the median value and the red cross denotes the mean value. The whiskers are lines that extend from the box to the highest and lowest values excluding outliers (o). Outliers represent those data with values being 1.5 higher or lower than the height of the box. Detailed data are given in supplementary data file.

Fig 5 shows the radionuclide ratios in surface water where pit lakes have been separated based on if they have been limed or not. The ^234^U/^238^U ratios are seen to be closer to unity and have a narrower range in limed lakes. A similar trend can be seen for ^210^Po/^238^U ratios where the rations are less spread compared to non-limed lakes where the interquartile range (25%-75%) varies with one order of magnitude.

### Chemical composition of sediments

**Fig 6:**
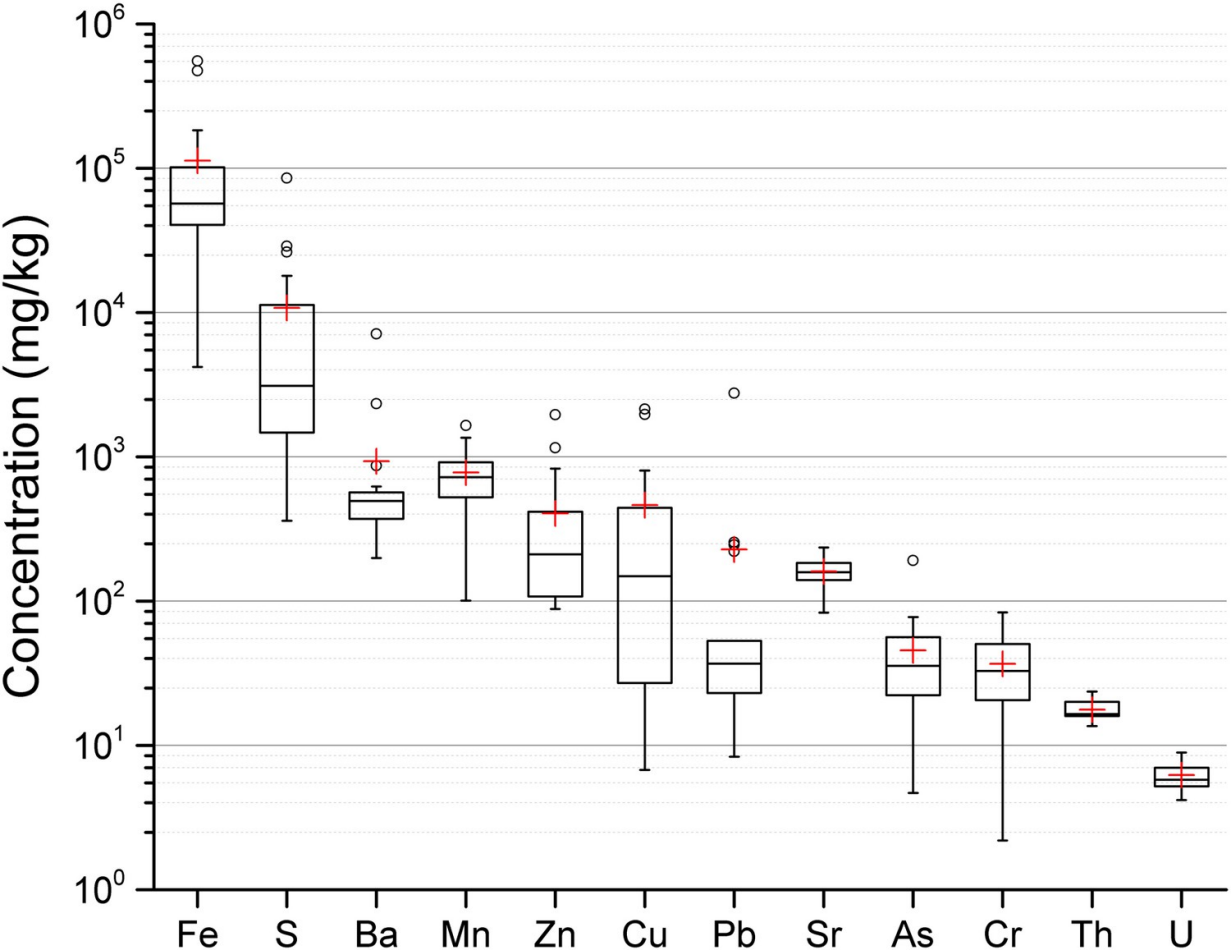
Element content per dry weight of surface sediment from 19 pit lakes, obtained by XRF analysis. The height of the box shows the interquartile range, which contains 50% of the values, the horizontal line inside the box shows the median value and the red cross denotes the mean value. The whiskers are lines that extend from the box to the highest and lowest values excluding outliers (o). Outliers represent those data with values being 1.5 higher or lower than the height of the box. Detailed data are given in supplementary data file.

The surface sediment samples from 19 pit lakes comprised mainly of SiO_2_ (6–92%), Fe_2_O_3_ (0.4–55%), Al_2_O_3_ (1.5–13%), K_2_O (0.4–4%), CaO (0.1–14%), Na_2_O (0.2–3%) and MgO (0.2–4%), with min-max levels in parenthesis. Other elements and their concentrations are shown in Fig 6. Due to similar ionic radius of Ca, Ba and Sr, both metals can replace Ca in crustal minerals [30]. For this reason, average concentration of Ba of 930 mg/kg and 140 mg/kg of Sr were observed in the sediments. The surface sediments collected at the shoreline seemed to be mostly minerogenic, i.e. composed of finely crushed minerals and rocks with little organic content, since the losses due to calcination were on average 5 ± 6% (SD). This could also be seen from Spearman’s correlation coefficients for the elements, where moderate to high correlations were found between elements that are known to co-exist in common minerals. For example, Ca and Mg for limestone (0.68), Al and Sr for feldspar (0.69), and Fe and S for pyrite (0.68). Furthermore, the precipitation of Fe minerals can also scavenge other elements from solution, such as As, Cu, Zn, Cr or Pb [31], e.g., the correlation coefficient between Fe and Cr in the present surface sediment was 0.77.

The highest correlation was between U and Th (0.87), where similar results were observed in previous studies of Swedish bedrock [32]. When comparing U and Th mass content in sediments, Th was about 3 times more abundant (Fig 6). However, this ratio was almost opposite in surface water, where Th was about 3 times less abundant (U: 1.5 μg/L and Th: 0.4 μg/L). These data are in agreement with previous studies and observations of the low solubility and high adsorption tendency of Th [4, 33].

However, the composition of sediments is strongly affected by local lithology. Therefore a comparison with background values is needed to confirm sediment pollution. In order to identify the metal enrichments in lake sediments studied, the methodology proposed by Håkanson [22] in Swedish lakes as a diagnostic tool for water pollution control was applied. In the present work, Hg and PCB concentrations were not determined and Cd was found below detection limit (0.1 ppm) in all sediments. Thus five metals were included in the assessment and the threshold values for Cd were adjusted accordingly (in steps of five instead of steps of eight). The sediments from some lakes showed very high contamination levels for Cu (Sites N4, N7A, N7B, N12 and N13), and to a lesser extent for Zn (Sites N4 and N13), Pb (Site N1B) and As (Site N12) (Table 1). In addition, most sediments suffer from moderate to considerable enrichment of As and Zn. However, most sediments seem to be unaffected or only slightly affected by Cr and Pb pollution. Although Fe was not included in this contamination factor, the high concentration of Fe in some sediment samples (e.g. N9 and N11) due to the enhanced transfer of Fe from the water column to the sediments by mining activities is also remarkable.

**Table 1:** Contamination factors (C_f_) and degree of contamination (C_d_) in sediments from 19 lakes assessed through a modified version of the model developed by Håkanson [22]. Cells with values for C_f_ <1 and C_d_ <5 are colored green, yellow for 1< Cf <3 and 5< Cd <10, orange for 3< Cf <6 and 10< Cd <20, and red for 6< Cf and 20< Cd.

	C_f_					C_d_
Site	As	Cu	Pb	Cr	Zn	
N1A	1.9	0.3	3.2	0.4	0.5	6.3
N1B	1.8	0.3	39.5	0.1	4.7	46.4
N2A	1.9	1.5	0.3	0.3	0.5	4.5
N3B	1.3	1.6	0.4	0.9	1.1	5.3
N4	2.3	8.9	0.8	0.7	6.6	19.2
N5	3.8	5.2	3.5	0.4	1.6	14.5
N6	0.3	0.1	0.1	0.1	0.6	1.2
N7A	2.8	16.1	0.5	0.1	1.4	20.7
N7B	3.8	42.8	0.6	0.2	2.4	49.8
N8	4.4	0.1	0.3	0.2	0.5	5.5
N9	0.1	0.1	0.1	0.1	0.1	0.3
N10B	2.5	0.5	0.7	0.3	3.8	7.8
N10C	1.2	0.1	0.2	0.0	1.0	2.5
N11A	1.1	0.1	0.2	0.3	1.0	2.6
N11B	1.5	0.1	0.1	0.5	1.1	3.1
N12	12.7	39.4	0.6	0.6	0.6	53.9
N13	3.1	3.5	0.5	0.6	11.2	18.9
N14	3.2	7.2	3.6	0.7	1.3	16.0
N15A	5.2	2.5	0.4	0.7	1.7	10.4

### Concentration ratios

**Fig 7:**
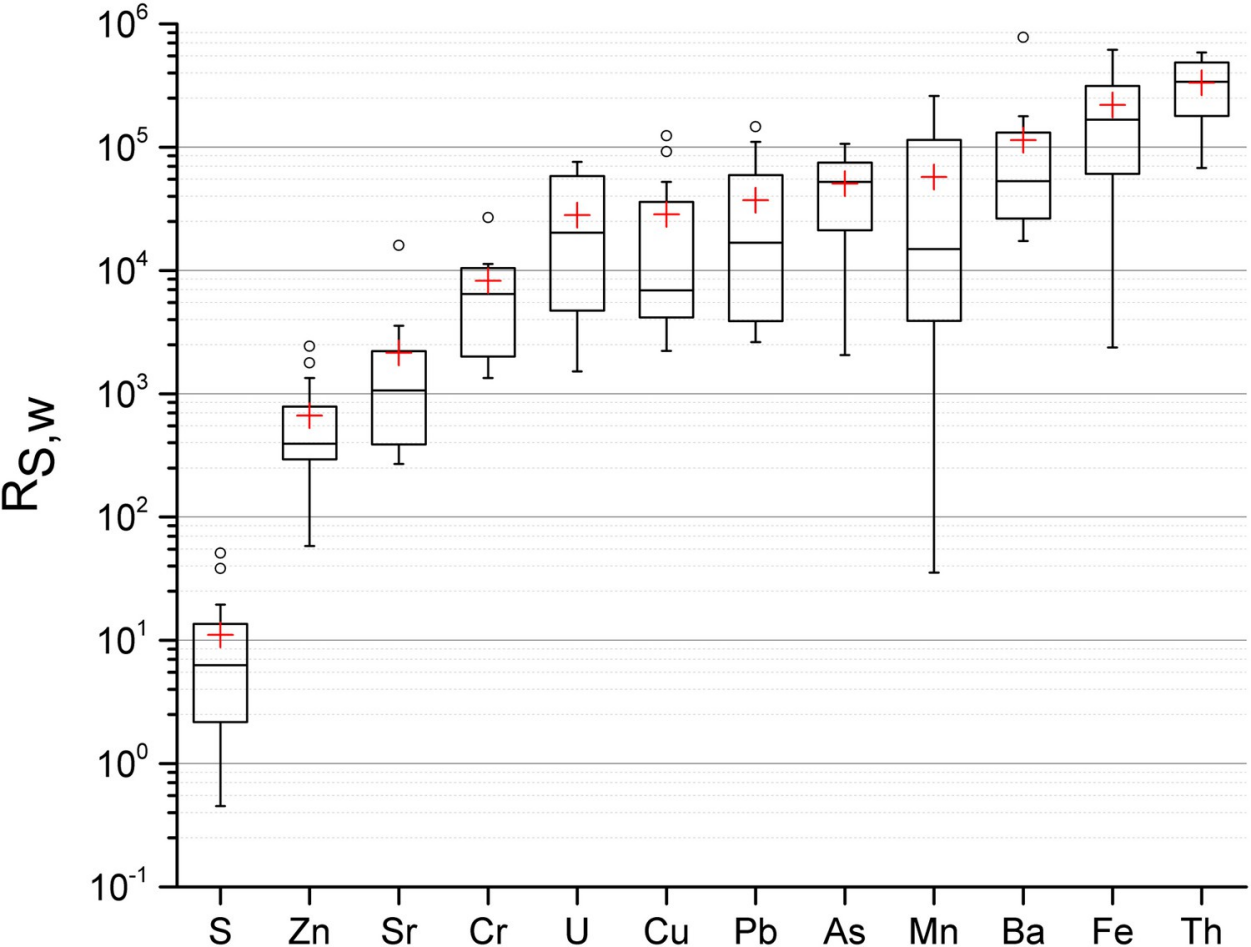
The ratio between element concentration in surface sediment and surface water, R_S,W_, from 19 pit lakes. The height of the box shows the interquartile range, which contains 50% of the values, the horizontal line inside the box shows the median value and the red cross denotes the mean value. The whiskers are lines that extend from the box to the highest and lowest values excluding outliers (o). Outliers represents those values being 1.5 larger than the length of the box from its upper or lower border. Data also given in supplementary data file.

R_S,W_ is defined as the ratio between element concentration in sediment and water. In some samples (waters or sediments) values close to or below the detection limit for some elements were observed, and as a consequence some R_S,W_ values are missing. Fig 7 presents R_S,W_ values for studied lakes. The lowest and highest median R_S,W_ value was found for S (6 L kg^-1^) and Th (3.4∙10^5^ L kg^-1^), respectively. It is also striking the higher R_S,W_ value observed for Th in relation to U, pointing out the low mobility of this radionuclide in aqueous media compared to U. If these ratios are compared with pit lakes from Southern Sweden [4], the median of the ratios for S, Cr, As, Mn, Fe, Th from Fig 7 are lower, except for Pb which is about one order of magnitude higher. The values of the remaining elements are within the same range. Since water and sediment were collected from the shoreline, these concentration ratios might not represent the lake as a whole. For example, sediments collected at accumulation sites would be less affected by water turbulence and erosion compared to these sediments collected from the shoreline.

### Radionuclides in sediments

The activity concentration in 18 sediment samples can be seen in Table 2. The activity concentrations of ^226^Ra, ^232^Th and ^40^K are within the ranges given by UNSCEAR [34]: 12–170 Bq/kg, 14–94 Bq/kg and 560–1150 Bq/kg, respectively. The results are also consistent with data for Swedish soils, reported by Evans and Eriksson [35]: about 70 Bq/kg for ^238^U and about 34 Bq/kg for ^232^Th.

**Table 2:** Activity concentration in Bq/kg in sediments with uncertainties given as 1σ. Results were obtained by gamma spectrometry, where ^232^Th was assumed to be in equilibrium with ^228^Ra (and ^228^Ac).

Site	^238^U series			^232^Th series	^137^Cs	^40^K
	^234^Th	^226^Ra	^210^Pb			
**N1A**	42±5	54±1	79±7	35±4	1.9±0.3	900±30
**N1B**	24±3	17±1	32±3	16±4	15.1±0.5	120±12
**N2A**	11±4	23±1	26±4	23±3	3.2±0.2	600±20
**N3B**	19±4	32±4	41±4	26±4	5.5±0.3	430±17
**N4**	29±4	45±2	72±4	33±4	1.6±0.3	780±26
**N5**	33±3	40±2	43±3	23±1	<0.78	740±23
**N6**	20±2	29±1	35±2	24±3	<0.5	910±28
**N7A**	36±5	41±2	91±10	30±3	11.9±0.5	840±27
**N7B**	29±4	44±2	120±4	33±2	16.4±0.6	890±29
**N8**	43±4	35±2	66±5	22±4	<1.35	810±27
**N9**	11±7	20±4	54±18	12±4	2.5±0.4	220±16
**N10B**	19±3	24±1	34±4	20±4	<1.08	860±28
**N10C**	40±4	32±4	30±4	19±2	2.9±0.3	730±25
**N11A**	15±3	18±1	41±3	14±4	<0.91	640±21
**N11B**	9±10	19±1	38±10	20±10	<2.5	350±27
**N12**	6±3	26±2	37±4	12±3	5.2±0.3	540±19
**N14**	18±3	28±1	40±6	19±2	<0.66	720±23
**N15A**	17±5	43±2	54±5	47±10	1.1±0.3	770±28

**Fig 8:**
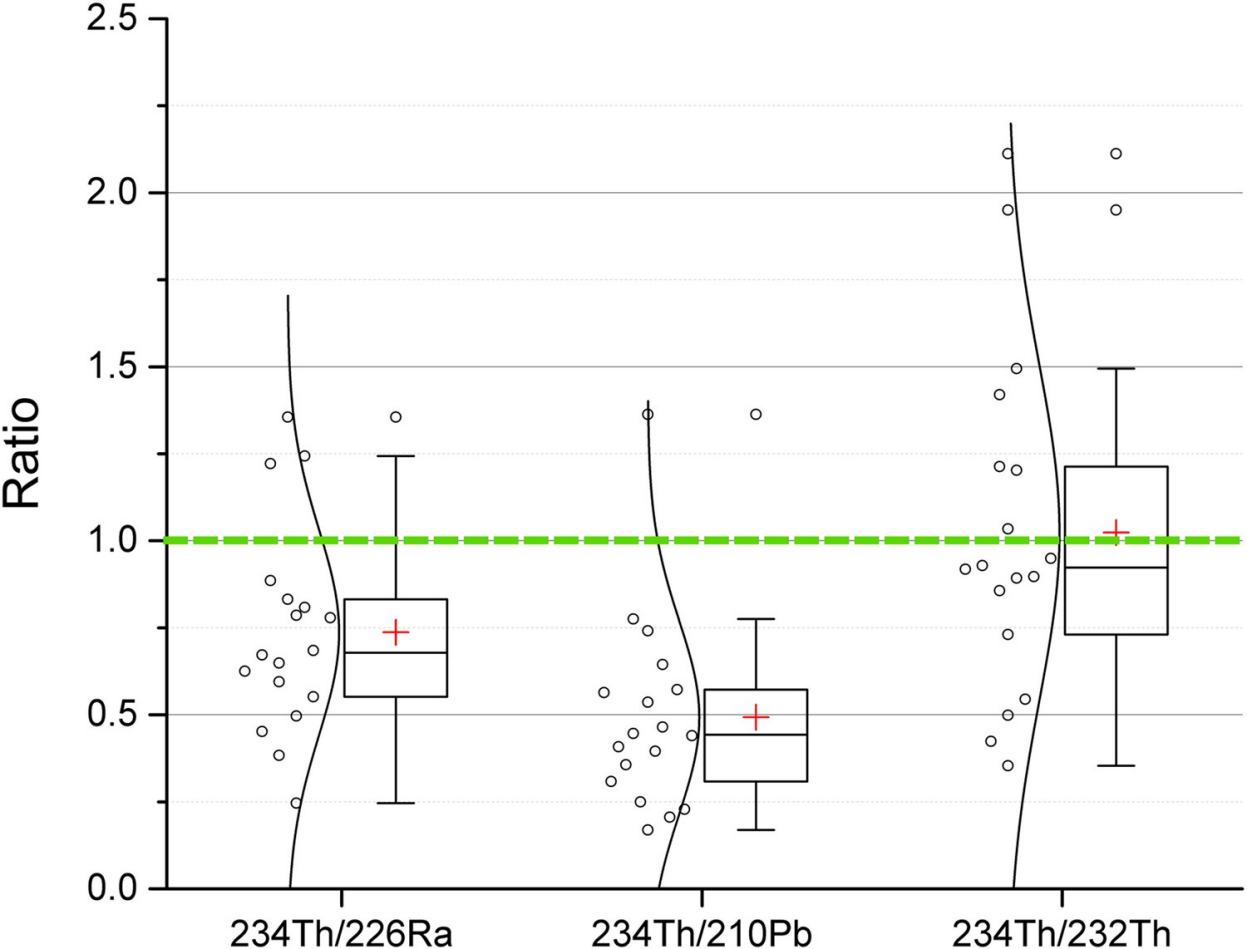
^234^Th/^232^Th, ^234^Th/^226^Ra and ^234^Th/^210^Pb activity concentration ratios in sediments from 18 pit lakes. Activity concentration was obtained by gamma spectrometry, where ^232^Th was assumed to be in equilibrium with ^228^Ra (and ^228^Ac). The ^234^Th/^226^Ra and ^234^Th/^210^Pb activity concentration ratios demonstrate disequilibrium in the ^238^U series (deviation from green line, ratio 1). Data are given as single values, as distribution of data points, and in box and whisker plots. The height of the box shows the interquartile range, which contains 50% of the values, the horizontal line inside the box shows the median value and the red cross denotes the mean value. The whiskers are lines that extend from the box to the highest and lowest values excluding outliers (o). Outliers represents those values being 1.5 larger than the length of the box from its upper or lower border. Data also given in supplementary data file.

Regarding the ^232^Th series, the activity concentration of ^228^Ac, ^212^Pb, ^212^Bi and ^208^Tl were in agreement within one coverage factor (K = 1). Thus, it was assumed that there was a secular equilibrium in the ^232^Th series, and the average of these radionuclides should be the activity concentration of ^232^Th. When studying the ^234^Th/^226^Ra and ^234^Th/^210^Po activity concentration ratios, a clear disequilibrium can be seen in several surface sediment samples, Fig 8. In general, the disequilibrium was larger the further down in the ^238^U series, with 0.74 ± 0.29 (mean ± SD) for ^234^Th/^226^Ra and 0.49±0.28 for ^234^Th/^210^Pb.

Regarding ^137^Cs in sediments, the majority of sediment samples (67%) from Northern Sweden had detectable concentrations with an average of 5.6 Bq/kg, whereas in Southern Sweden the average was 2.8 Bq/kg with 62% of samples having concentrations above MDA [4]. These findings are in agreement with the deposition distribution within Sweden after the Chernobyl accident [36].

## Conclusions

The results from this screening study showed that pH values in surface water were mostly neutral, and specific conductivity levels were normal compared to other studies of groundwater in Sweden. Enhanced concentration of trace metals was observed in lakes water due to mining. The concentration of elements in surface water was not always reflecting the type of mine or mineral deposits at the site. For example, Zn in surface water could range from 0.1 to 32 mg/L in former Zn mines. In general, the concentration in surface water varied with only about one order of magnitude between all the pit lakes studied. On the other hand, the surface sediments showed a much larger variation between the pit lakes and the metals with relatively high concentrations were the same metals that were mined at the sites.

The concentration of radionuclides in both surface water and sediment were relatively low in Northern Sweden, compared to Southern Sweden. Activity concentration in surface water and sediment, as well as the ambient dose equivalent rate was similar to background levels in Sweden. Disequilibrium was found for the ^238^U series, both in surface water and surface sediments, that increased further down in the decay series. This disequilibrium was most pronounced in surface water.

In conclusion, in contrast to previously studied pit lakes from Southern Sweden, sites from Northern Sweden do not pose any radiological risk for the environment through naturally occurring radionuclides. However, from a trace element contamination perspective, several sites were identified having enhanced concentrations in need to be addressed.

## Supporting information

S1 File (PDF)
