# Supplementary material for: Natural radioactivity and element characterization in pit lakes in Northern Sweden
Source: PLoS One. 2022 Mar 31;17(3):e0266002. doi: 10.1371/journal.pone.0266002 (PMC8970354; doi:10.1371/journal.pone.0266002)
Supplement: S1 File — (PDF) [file pone.0266002.s001.pdf]

Supplementary data

Natural radioactivity and element characterization in pit lakes in Northern Sweden

Rimon Thomas[1*], Juan Mantero[1,2], Carlos Ruiz Cánovas[3], Elis Holm[1†], Rafael García-Tenorio[2,4], Eva Forssell-Aronsson[1,5] & Mats Isaksson[1]

[1]Department of Medical Radiation Sciences, Institute of Clinical Sciences, Sahlgrenska Academy at University of Gothenburg, 41345 Gothenburg, Sweden

[2]Department of Applied Physics II, ETSA, University of Seville, Seville, 41012, Spain

[3]Department of Earth Sciences & Research Center on Natural Resources, Health and the Environment. University of Huelva, Huelva, 21071, Spain.

[4]Spanish National Accelerator Centre (CNA), University of Seville, 41092, Spain

[5]Department of Medical Physics and Biomedical Engineering, Sahlgrenska University Hospital, Region Västra Götaland, 41345 Gothenburg, Sweden

*E-mail: rimon.thomas@gu.se

Supplementary Table S1 Description of the mines studied including name of the mine, coordinates, years of operation, main metal(s) extracted, approximate volume of the pit lake and if the lake is or has been included in a liming program

Supplementary Table S2 pH, specific conductance and total dissolved solids in surface water from the studied pit lakes, and ambient dose equivalent rate measured around the pit lake at 1 m height

Supplementary Table S3 Concentration of various elements in surface water samples from the studied pit lakes. Values were obtained by ICP-MS analysis and uncertainties are in the range 10% to 20% (k=1). ND=Not detected

Supplementary Table S4 Activity concentration of ^{210}Po, U and Th isotopes in mBq/kg in surface water samples from pit lakes determined by alpha spectrometry. NM indicates the radionuclide was not measured in the sample. Data are given as activity concentration ± uncertainty (k=1)

Supplementary Table S5 Concentration and composition of elements in surface sediments from pit lakes, analyzed by XRF. Data are presented as percentage (%) or in ppm. ND=Not detected, indicates that the concentration of the element was below detection limit (1-5 ppm)

Photographs of pit lakes (taken by Juan Mantero)

Supplementary data

Table S1 Description of the mines studied including name of the mine, years of operation, main metal(s) extracted, approximate volume of the pit lake and if the lake is or has been included in a liming program

Site	Name of the mine	Coordinates WGS84	Years of operation	Metal	Volume [m^3]	Limed
**N1**	Vassbogruvan	61.939596, 12.490082	1985-1986	Pb, Zn	Unknown	No
**N2**	Enåsengruvan	62.286283, 15.370416	1984-1991	Cu, Au	500000	Yes
**N3**	Stekenjokkgruvan	65.099935, 14.461707	1976-1989	Cu, Zn	Unknown	No
**N4**	Rävlidmyrgruvan	65.063847, 18.477457	1977-1989	Zn	527000	Yes
**N5**	Rävlidengruvan	65.078957, 18.472020	1977-1989	Zn	61900	Yes
**N6**	Näslidengruvan	65.099442, 19.103046	1962-1989	Cu, Zn	134000	No
**N7**	Lavergruvan	65.771924, 20.239657	1938-1947	Cu	Unknown	No
**N8**	Åkerbergsgruvan	65.042422, 20.744890	1989-1901	Au	300000	No
**N9**		64.872527, 20.378741	1924-1967	Au, Cu	Unknown	No
**N10**	Långdalgruvan	64.846665, 20.276068	1966-1999	Zn, Au	1110000	Yes
**N11**	Långselegruvan	64.825805, 20.227654	1957-1990	Zn, Cu	57500	No
**N12**	Åkullagruvan (Västra)	64.907414, 20.250694	1946-1957	Cu, Au	137000	Yes
**N13**	Uddengruvan	64.958079, 19.837617	1970-1989	Cu, Zn	563000	Yes
**N14**	Åsengruvan	64.937357, 19.880607	1970, 1988-1994	Cu, Zn	81500	Yes
**N15**	Kedträskgruvan	64.950864, 19.885618	1969-1972, 1988-1990	Cu, Zn	450000	Yes
**N16**	(Kiruna region)	67.892342, 20.255118	Unknown			
**N17**	(Kiruna region)	67.898524, 20.260022	Unknown			

Supplementary data

Table S2 pH and specific conductance (corrected to 25ºC) in surface water from the studied pit lakes, and ambient dose equivalent rate measured around the pit lake at 1 m height

Sample	pH	Specific conductance (µS/cm)	Ambient dose equivalent rate [µSv/h]
**WN1A**	7.26	100	0.14
**WN1B**	9.33	150	0.11
**WN2A**	8.17	840	0.13
**WN2B**	7.79	830	0.13
**WN3A**	8.04	30	0.09
**WN3B**	7.40	300	0.10
**WN4**	5.73	500	0.11
**WN5**	8.98	1700	0.10
**WN6**	8.59	170	0.10
**WN7A**	7.28	40	0.12
**WN7B**	6.91	90	0.12
**WN8**	7.79	120	0.08
**WN9A**	2.53	3200	0.09
**WN9B**	3.00	2200	0.09
**WN10A**	6.92	40	0.10
**WN10B**	8.22	670	0.11
**WN10C**	6.99	40	0.09
**WN11A**	8.44	960	0.09
**WN11B**	5.94	3800	0.07
**WN12**	9.21	700	0.08
**WN13A**	7.2	1900	0.08
**WN13B**	7.12	1900	0.09
**WN14**	6.75	250	0.11
**WN15A**	6.95	1100	0.09
**WN15B**	6.85	1100	0.10
**WN16**	7.55	130	0.11
**WN17**	7.69	100	0.08

Supplementary data

Table S3 Concentration of various elements in surface water samples from the studied pit lakes. Values were obtained by ICP-MS analysis and uncertainties are in the range 10% to 20% (k=1). ND=Not detected

	**mg/L**							**µg/L**								**ng/L**
**Sample**	**Na**	**Mg**	**S**	**K**	**Ca**	**Fe**	**Mn**	**Cr**	**Cu**	**Zn**	**As**	**Sr**	**Ba**	**Pb**	**U**	**Th**
**WN1A**	2.8	10	110	8.3	32	ND	2.4	ND	5.3	120	0.7	45	34	3.7	0.2	220
**WN1B**	4.4	2.7	340	4.5	90	ND	26	6.1	4.0	340	0.4	130	263	80	0.2	72
**WN2A**	12	12	2400	46	500	ND	52	ND	18	220	0.4	310	11	3.4	0.4	ND
**WN2B**	19	20	2400	28	380	0.9	40	3.4	12	230	ND	330	6.7	2.4	0.4	ND
**WN3A**	ND	ND	24	ND	2.5	0.1	ND	11	60	ND	ND	3.4	ND	ND	ND	ND
**WN3B**	7.3	8.6	91	9.1	130	ND	52	ND	17	510	0.4	270	2.9	5.0	1.5	ND
**WN4**	ND	0.8	319	2.5	62	ND	170	12	130	3900	ND	120	ND	ND	ND	ND
**WN5**	26	190	4200	69	630	ND	7.2	4.6	7.2	540	ND	600	4.9	1.7	ND	ND
**WN6**	10	13	110	3.3	60	ND	ND	ND	4.1	80	ND	81	6.4	2.2	1.2	ND
**WN7A**	ND	ND	39	0.7	2.2	0.1	ND	13	88	210	ND	12	ND	ND	ND	ND
**WN7B**	14	4.7	ND	24	18	0.1	30	ND	17	1100	ND	52	0.6	0.4	ND	ND
**WN8**	9.5	19	370	6.6	92	0.5	3.1	8.0	3.0	240	32	190	7.8	5.3	4.2	ND
**WN9A**	71	230	6800	64	740	240	6500	17	4800	32000	8.4	590	6.3	380	2.0	890
**WN9B**	22	130	6300	17	450	67	6200	22	6300	30000	2.2	240	8.3	23	6.8	1800
**WN10A**	1.9	1.3	ND	ND	55	0.2	6.3	2.6	0.9	ND	ND	36	2.0	2.9	ND	ND

Supplementary data

Table S3 continued

	mg/L							µg/L								ng/L
**Sample**	**Na**	**Mg**	**S**	**K**	**Ca**	**Fe**	**Mn**	**Cr**	**Cu**	**Zn**	**As**	**Sr**	**Ba**	**Pb**	**U**	**Th**
**WN10B**	16	30	970	41	350	0.2	120	3.1	5.7	940	0.4	310	11	5.5	1.0	36
**WN10C**	ND	0.2	38	1.4	5.9	0.1	ND	11	ND	320	ND	17	0.3	ND	ND	ND
**WN11A**	15	28	2300	52	580	0.3	4.5	ND	7.1	400	ND	390	16	5.9	ND	ND
**WN11B**	64	480	8600	320	1300	200	8400	ND	17	3400	9.1	900	11	12	1.4	71
**WN12**	23	16	1300	26	410	0.6	32	ND	21	330	ND	370	3.8	1.0	0.2	36
**WN13A**	18	26	4200	58	1300	ND	150	1.9	5.6	1100	ND	580	10	1.8	0.4	ND
**WN13B**	25	36	5900	41	1000	0.01	160	1.3	5.6	890	4.2	610	8.1	2.9	ND	ND
**WN14**	8.8	12	800	13	100	0.6	170	5.8	78	1000	ND	85	17	3.0	0.1	ND
**WN15A**	13	39	2800	21	640	ND	1000	ND	2.4	1200	0.7	310	13	2.7	0.1	ND
**WN15B**	20	53	4300	12	580	0.4	870	ND	3.9	1500	ND	350	16	2.5	1.0	ND
**WN16**	0.6	0.6	ND	1.5	7.1	0.08	5.8	20	ND	36	ND	48	23	33	4.1	ND
**WN17**	0.7	0.9	ND	2.3	8.6	0.06	5.8	4.6	12	160	ND	29	21	56	1.4	ND

Supplementary data

Table S4 Activity concentration of ^{210}Po, U and Th isotopes in mBq/kg in surface water samples from pit lakes determined by alpha spectrometry. NM indicates the radionuclide was not measured in the sample. Data are given as activity concentration ± uncertainty (k=1)

Sample	^{238}U	^{234}U	^{230}Th	^{210}Po	^{232}Th
**W N1A**	1.9±0.2	2.3±0.2	<0.6	3.4±0.8	<0.4
**W N1B**	2.1±0.3	3.8±0.4	<0.7	5.0±0.6	<0.4
**W N2A**	6.6±0.8	10.0±1.0	<0.9	1.0±0.2	<0.6
**W N2B**	6.6±0.5	8.4±0.6	<0.8	NM	<0.5
**W N3A**	0.3±0.1	0.3±0.2	<0.1	12.6±0.8	<0.1
**W N3B**	18.5±0.9	24±1	<1.2	3.4±0.5	<0.8
**W N4**	4.4±0.5	6.5±0.6	<0.7	3.3±0.6	<0.4
**W N5**	1.0±0.3	1.1±0.3	<1.1	1.4±0.3	<0.7
**W N6**	3.6±0.4	8.4±0.6	<0.8	3.0±0.4	<0.5
**W N7A**	NM	NM	<0.2	10.8±0.8	<0.2
**W N7B**	3.8±0.7	6.1±0.6	<0.3	1.0±0.2	<0.2
**W N8**	85±5	161±8	<0.9	4.2±0.6	<0.6
**W N9A**	73±3	92±3	5.5±0.7	17±1	2.9±0.4
**W N9B**	NM	NM	12.0±1.3	15±1	8.9±0.8
**W N10A**	11.8±1.0	19±1	<0.3	4.0±0.4	<0.2
**W N10B**	NM	NM	<1.1	2.2±0.5	<0.8
**W N10C**	1.6±0.2	2.9±0.3	<0.9	3.7±0.5	<0.6
**W N11A**	1.0±0.1	1.2±0.1	<0.7	2.2±0.4	<0.4
**W N11B**	13.9±1.4	19±2	<0.5	2.7±0.4	<0.3
**W N12**	1.0±0.3	1.3±0.3	<0.5	16±1	<0.5
**W N13A**	2.3±0.4	3.0±0.4	<0.5	1.0±0.3	<0.4
**W N13B**	2.7±0.5	3.4±0.4	<0.6	1.4±0.2	<0.4
**W N14**	1.4±0.4	2.0±0.4	<0.7	2.7±0.5	<0.5
**W N15A**	1.0±0.3	1.2±0.3	<0.7	2.3±0.4	<0.5
**W N15B**	1.4±0.2	1.0±0.2	<0.8	3.1±0.4	<0.5
**W N16A**	56±3	109±5	<0.5	1.6±0.7	<0.3
**W N17A**	16±3	31±3	<0.7	3.1±0.6	<0.5

Supplementary data

Table S5 Concentration and composition of elements in surface sediments from pit lakes, analyzed by XRF. Data are presented as percentage (%) or in ppm. ND=Not detected, indicates that the concentration of the element was below detection limit (1-5 ppm)

**Sample**	**SiO_2 [%]**	**Al_2O_3 [%]**	**Fe_2O_3 [%]**	**MnO [%]**	**SO_3 [%]**	**As [ppm]**	**Ba [ppm]**	**Cr [ppm]**	**Cu [ppm]**	**Pb [ppm]**	**Sr [ppm]**	**Th [ppm]**	**U [ppm]**	**Zn [ppm]**
**Sed N1A**	73	9.5	5.3	0.03	0.6	29	870	33	13	220	102	15	4.2	94
**Sed N1B**	92	2.4	0.4	0.01	0.3	26	7100	8.2	17	2800	139	21	8.9	830
**Sed N2**	60	8.3	4.1	0.08	0.5	29	290	31	74	23	83	24	8.6	88
**Sed N3**	64	12	8.1	0.14	0.1	19	300	84	78	28	190	14	5.1	200
**Sed N4**	63	12	10	0.09	0.3	34	500	61	440	53	180	16	5.2	1200
**Sed N5**	63	11	5.7	0.08	0.2	56	620	35	260	250	170	16	5.0	290
**Sed N6**	72	12	4.2	0.07	0.04	4.7	380	2.2	ND	8.4	160	18	5.7	110
**Sed N7A**	72	12	4.1	0.07	0.15	42	510	ND	800	32	190	17	6.2	250
**Sed N7B**	70	10	6.1	0.13	0.3	58	490	20	2100	40	170	17	6.6	420
**Sed N8**	68	10	3.2	0.05	0.06	65	450	21	6.8	18	240	16	6.4	89
**Sed N9**	5.6	1.5	55.4	0.02	8.6	ND	ND	ND	ND	ND	ND	ND	ND	ND
**Sed N10A**	72	9.6	5.6	0.16	0.13	37	360	25	27	48	130	22	8.1	670

Supplementary data

Table S5 continued

Sample	SiO_2 [%]	Al_2O_3 [%]	Fe_2O_3 [%]	MnO [%]	SO_3 [%]	As [ppm]	Ba [ppm]	Cr [ppm]	Cu [ppm]	Pb [ppm]	Sr [ppm]	Th [ppm]	U [ppm]	Zn [ppm]
**Sed N10B**	75	9.6	3.6	0.08	0.2	19	390	4.5	ND	17	150	21	7.7	170
**Sed N11A**	74	11	4.0	0.07	0.2	16	370	28	ND	15	150	20	7.0	180
**Sed N11B**	22	3.0	47	0.05	2.9	22	200	43	ND	ND	ND	ND	ND	200
**Sed N12**	56	7.5	18	0.07	2.6	190	510	50	2000	44	140	14	4.8	100
**Sed N13**	65	13	7.2	0.13	0.3	47	510	51	170	37	160	16	5.8	2000
**Sed N14**	66	11	9.5	0.06	1.1	48	2300	66	360	260	190	17	5.4	220
**Sed N15**	59	10	13	0.08	1.8	78	570	63	120	29	180	17	5.3	290

Supplementary data

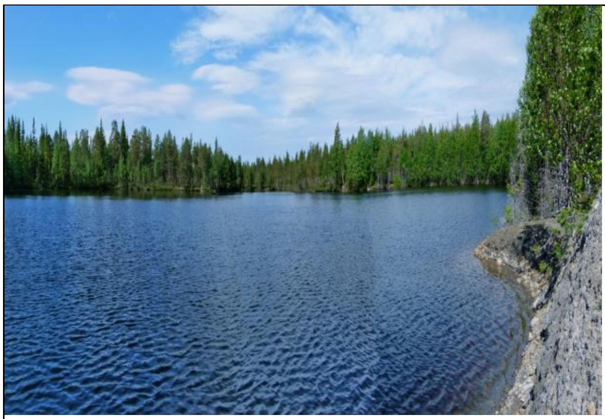

Site N1A

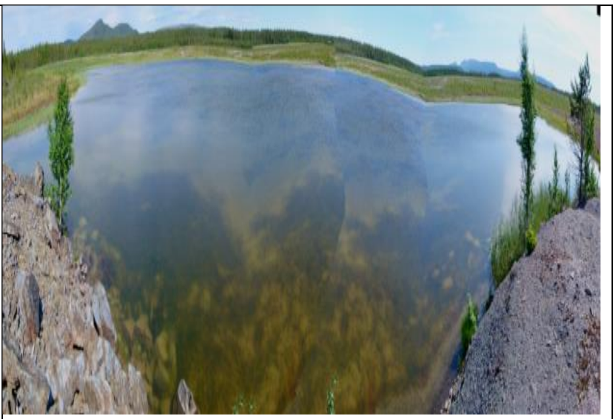

Site N1B

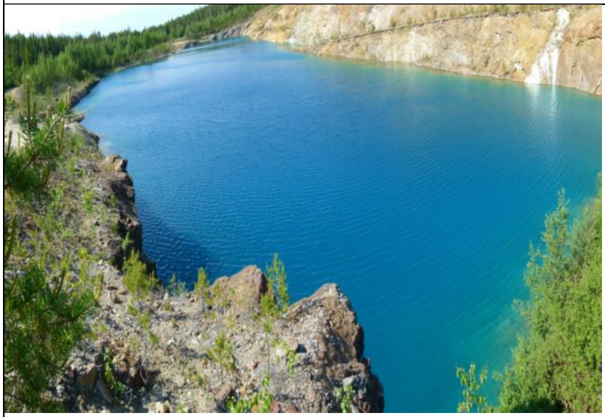

Site N2

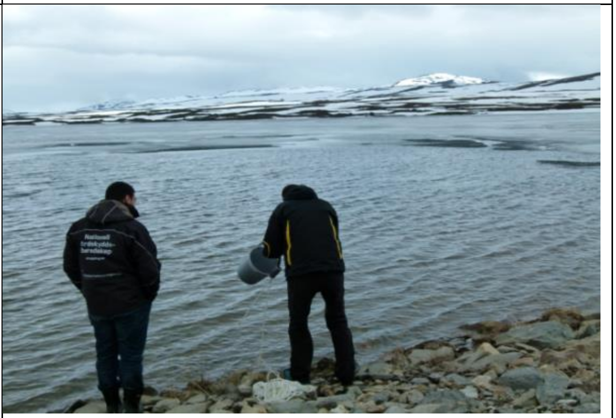

Site N3A

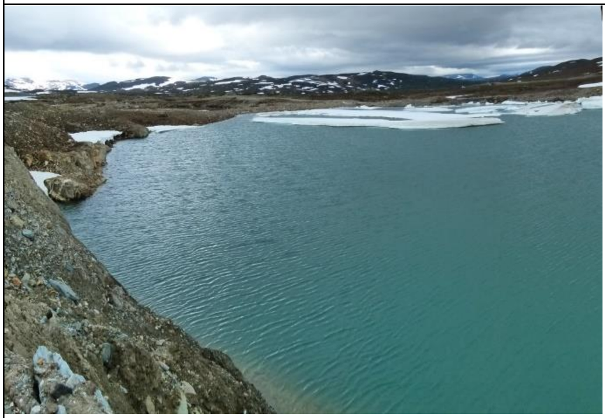

Site N3B

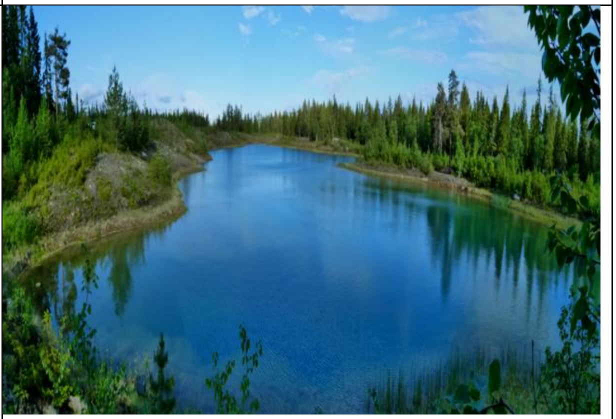

Site N4

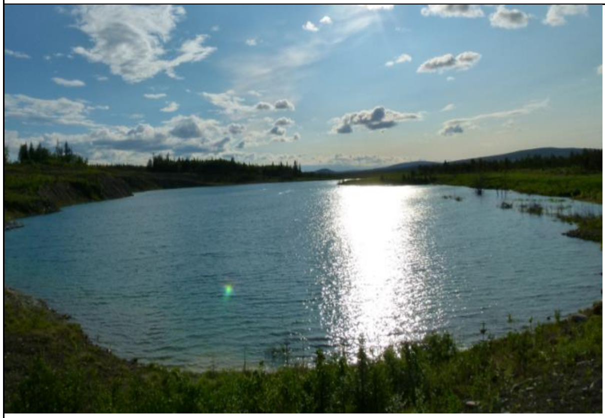

Site N5

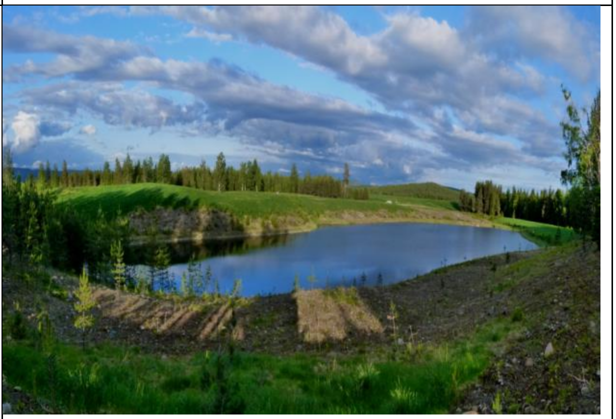

Site N6

Supplementary data

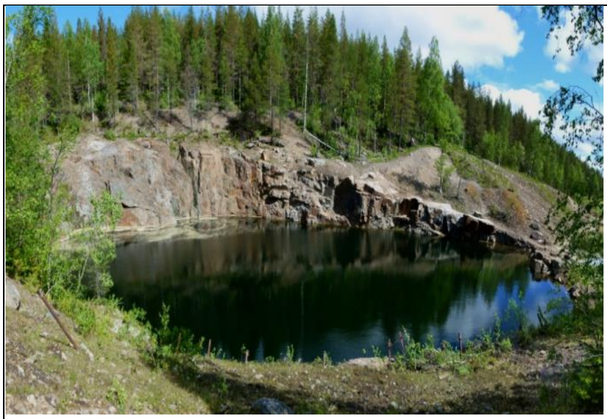
Site N7A

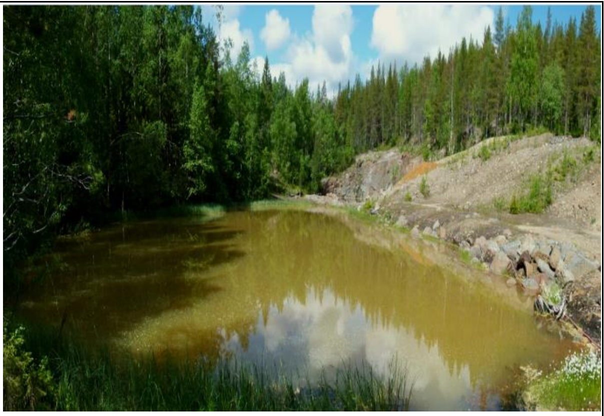
Site N7B

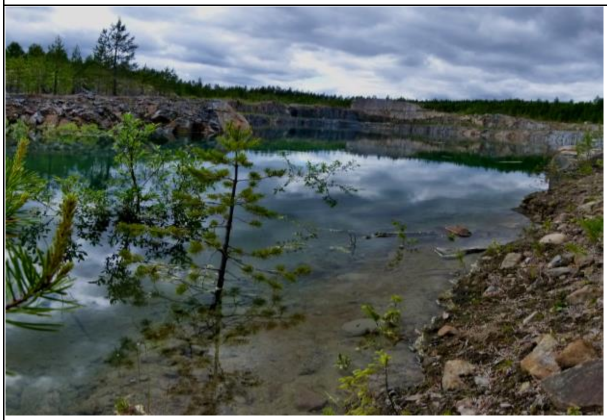
Site N8

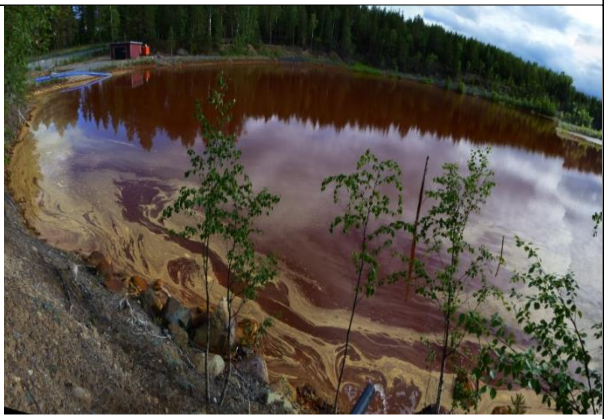
Site N9

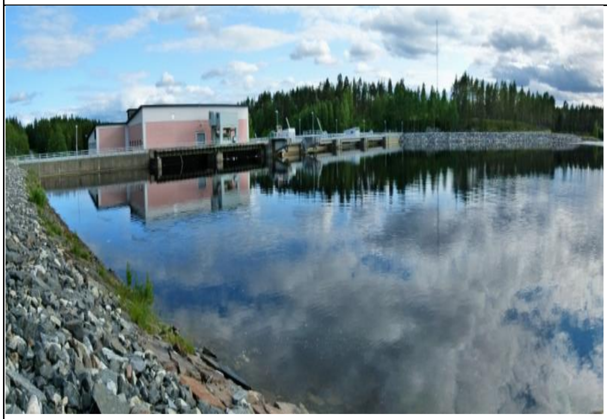
Site N10A

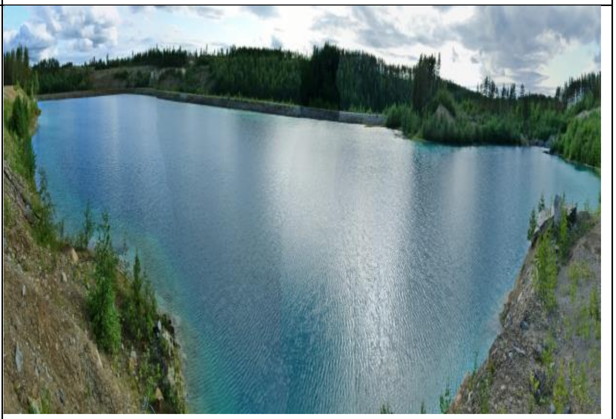
Site N10B

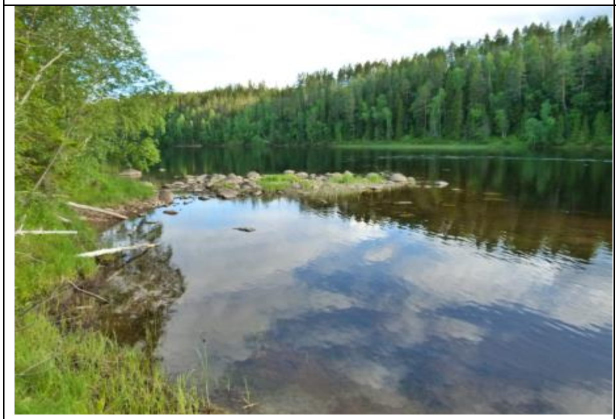
Site N10C

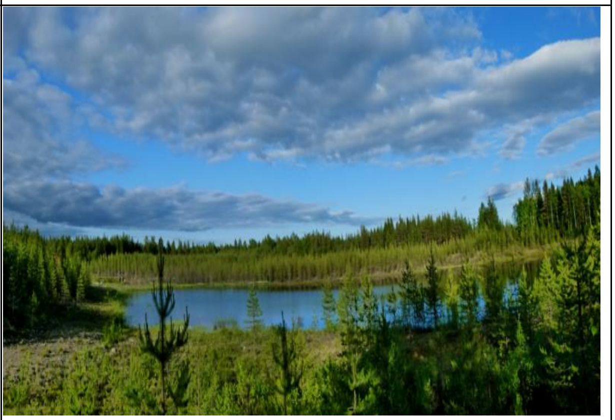
Site N11A

Supplementary data

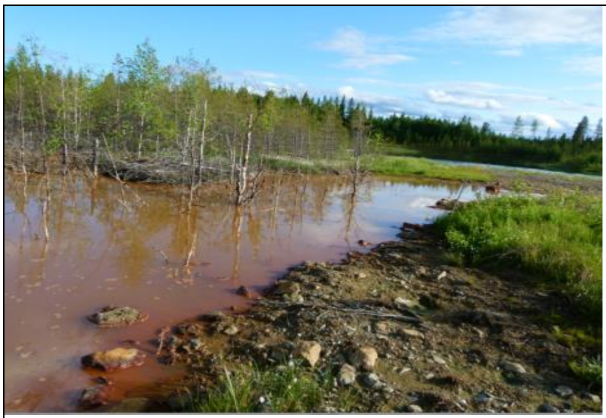

Site N11B

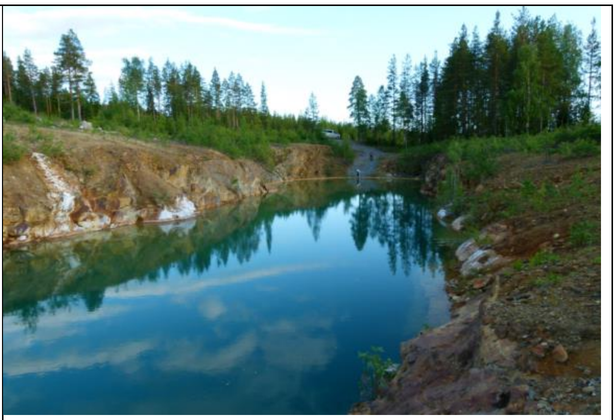

Site N12

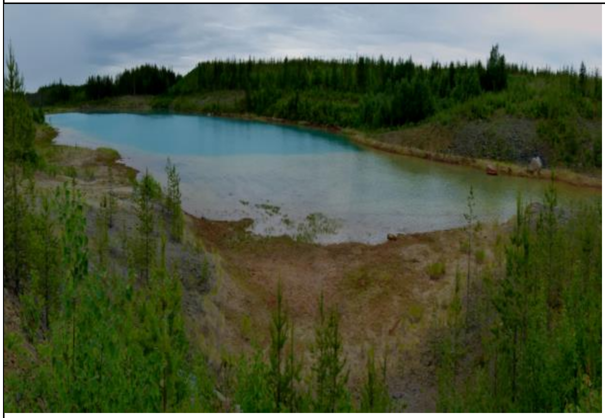

Site N13A

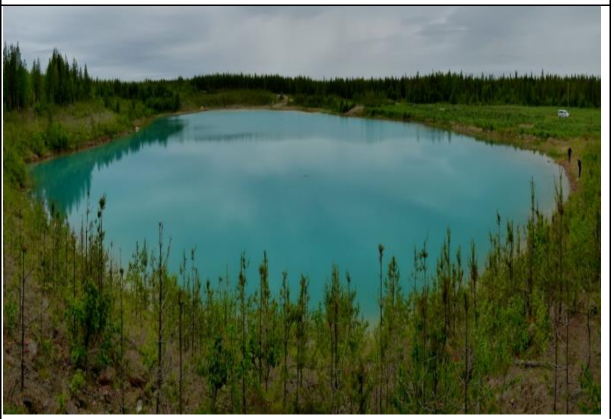

Site N13B

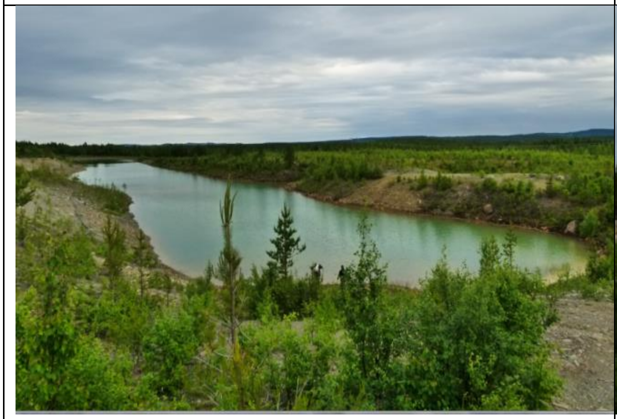

Site N14

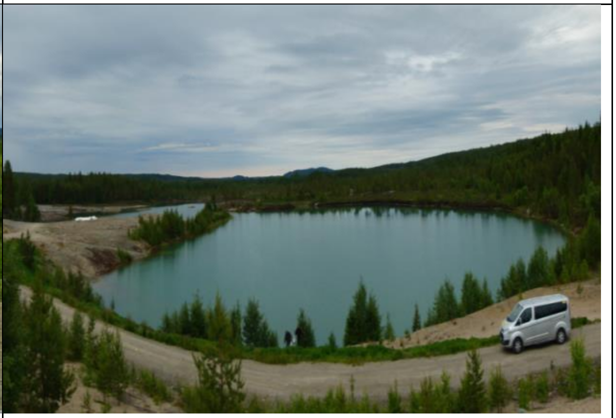

Site N15A

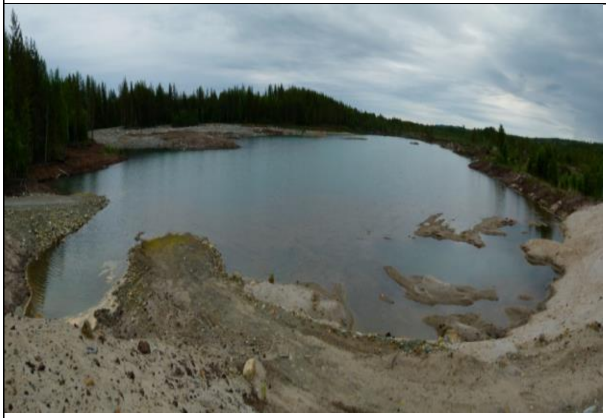

Site N15B

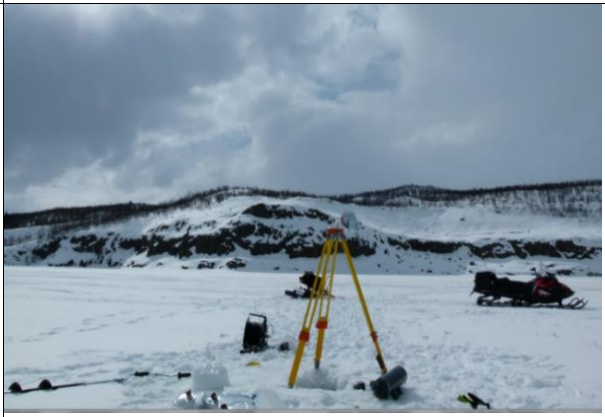

Site N16

Supplementary data

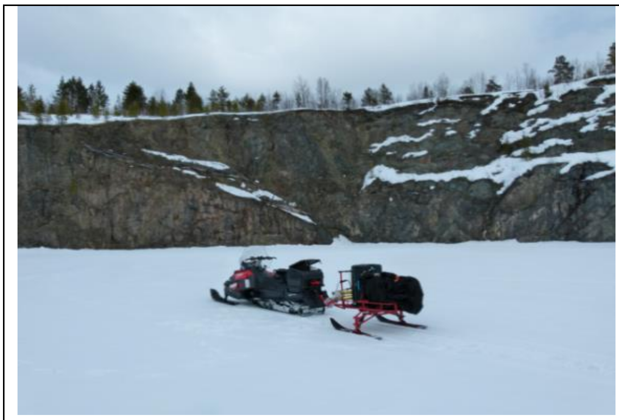

Site N17
